# Brain Edema Formation and Functional Outcome After Surgical Decompression in Murine Closed Head Injury Are Modulated by Acetazolamide Administration

**DOI:** 10.3389/fneur.2019.00273

**Published:** 2019-03-26

**Authors:** Jacek Szczygielski, Vanessa Hubertus, Eduard Kruchten, Andreas Müller, Lisa Franziska Albrecht, Angelika E. Mautes, Karsten Schwerdtfeger, Joachim Oertel

**Affiliations:** ^1^Department of Neurosurgery, Saarland University Medical Center and Saarland University Faculty of Medicine, Homburg, Germany; ^2^Institute of Neuropathology, Saarland University Medical Center and Saarland University Faculty of Medicine, Homburg, Germany; ^3^Faculty of Medicine, University of Rzeszów, Rzeszów, Poland; ^4^Department of Neurosurgery, Charité University Medicine, Berlin, Germany; ^5^Institute of Interventional and Diagnostic Radiology, Karlsruhe Municipal Hospital, Karlsruhe, Germany; ^6^Department of Radiology, Saarland University Medical Center and Saarland University Faculty of Medicine, Homburg, Germany

**Keywords:** traumatic brain injury, acetazolamide, decompressive craniectomy, brain edema, mouse, closed head injury

## Abstract

Acetazolamide (ACZ), carbonic anhydrase inhibitor, has been successfully applied in several neurosurgical conditions for diagnostic or therapeutic purposes. Furthermore, neuroprotective and anti-edematous properties of ACZ have been postulated. However, its use in traumatic brain injury (TBI) is limited, since ACZ-caused vasodilatation according to the Monro-Kellie doctrine may lead to increased intracranial blood volume / raise of intracranial pressure. We hypothesized that these negative effects of ACZ will be reduced or prevented, if the drug is administered after already performed decompression. To test this hypothesis, we used a mouse model of closed head injury (CHI) and decompressive craniectomy (DC). Mice were assigned into following experimental groups: sham, DC, CHI, CHI+ACZ, CHI+DC, and CHI+DC+ACZ (*n* = 8 each group). 1d and 3d post injury, the neurological function was assessed according to Neurological Severity Score (NSS) and Beam Balance Score (BBS). At the same time points, brain edema was quantified by MRI investigations. Functional impairment and edema volume were compared between groups and over time. Among the animals without skull decompression, the group additionally treated with acetazolamide demonstrated the most severe functional impairment. This pattern was reversed among the mice with decompressive craniectomy: CHI+DC treated but not CHI+DC+ACZ treated animals showed a significant neurological deficit. Accordingly, radiological assessment revealed most severe edema formation in the CHI+DC group while in CHI+DC+ACZ animals, volume of brain edema did not differ from DC-only animals. In our CHI model, the response to acetazolamide treatment varies between animals with decompressive craniectomy and those without surgical treatment. Opening the cranial vault potentially creates an opportunity for acetazolamide to exert its beneficial effects while vasodilatation-related risks are attenuated. Therefore, we recommend further exploration of this potentially beneficial drug in translational research projects.

## Introduction

Acetazolamide (ACZ), a sulfonamide derivate is widely used in neuroscience research as well as in clinical practice. In the central nervous system multiple mechanisms of action have been described. Due to non-competitive carbonic anhydrase inhibition, ACZ reduces ${\text{HCO}}_{3}^{-}$ concentration, leading to pH-mediated vasodilation of cerebral vessels (1) and reduced secretion of cerebrospinal fluid (CSF) (2). ACZ also modulates the action of aquaporins (AQP) including AQP4 (3–8), the main water channel protein involved in brain edema formation (9). In addition to direct modulation of AQP function, the diuretic effect of ACZ may be responsible for restricting brain edema formation. One target of ACZ action is the proximal renal tubule (10). By reducing the sodium and bicarbonate reabsorption, moderate water loss is induced (11), and this effect is projected also on the brain water content (12, 13). Additionally, neuroprotective properties of acetazolamide have been described (3, 14). As a possible mechanism of action a modulation of ion channel function has been proposed (14).

Some of these properties have been successfully integrated into clinical practice, to decrease intracranial pressure (ICP) via reduced CSF production in benign intracranial hypertension (15, 16). Also the ACZ-mediated vasodilation has been utilized for treatment of acute mountain disease (17) and for diagnostic neuroimaging (18–22). Furthermore, in traumatic brain injury (TBI) experiments, antiedematous, and neuroprotective effects of ACZ treatment could be demonstrated, and its therapeutic potential has been postulated (3, 6, 7). While ACZ has been applied for detection of impaired vascular autoregulation in mild TBI (23), the vasoactive effect of ACZ may play also negative role in treatment of severe head injury: ACZ increases intracranial blood volume and may therefore, according to Monro-Kellie doctrine, cause ICP increase (17, 24, 25).

However, decompressive craniectomy (DC)—performed e.g., for massive brain swelling or acute subdural hematoma—may serve as a preventive countermeasure, since this surgical technique leads to immediate decrease of ICP and adds an extra compensatory space for intracranial volume (26). Nevertheless, the effects of craniectomy as a treatment of severe TBI are still matter of debate. Certainly, DC is a life-saving procedure, as critically high ICP results in poor outcome (27, 28). However, mechanical decompression may cause additional brain damage (29–32) and does not necessarily improve patients‘ outcome (33). Therefore, if craniectomy is necessary for ICP reduction (34, 35), effort should be undertaken in order to blunt its potential negative impact. In this context ACZ is a good candidate drug for combination with craniectomy treatment. ACZ was already used in clinical conditions related to TBI and seems to be reasonably safe (24, 36–38); it has also been shown to exert neuroprotective effect (14). ACZ also beneficially affects water turnover in intracranial compartments (39).

In light of the extensive and complex evidence of ACZ actions, it is hard to predict, whether its lone use or application as a supplementary drug after surgical decompression would have a positive or negative effect on outcome after severe TBI. To test the net effect of ACZ in experimental setting, the head injury plus craniectomy model must resemble the sequence that is experienced by TBI patients. Recently, based on the closed head injury paradigm, we have establish an experimental design which fulfills this requirement (31).

In our current study, we test the hypothesis that the impact of ACZ on the posttraumatic functional outcome and brain edema formation differs between subjects treated with and without skull decompression. To test this hypothesis, we implemented ACZ administration in our murine model of DC after closed head injury (CHI).

## Methods

### Animals and Trauma Model and Administration of ACZ

All animal experiments were performed with approval by the local ethical board (18/2013, Saarland Ethical Commission), in line with the laws for animal protection, including Directive 2010/63/EU and by following all institutional and national guidelines for the care and use of laboratory animals.

Male wild-type, CD-1 mice of 9–12 weeks of age naïve to previous surgical or drug treatment weighting 37.2 ± 0.9 g were acquired from the Charles River Laboratories South Germany and kept in local Animal Facility, housed in groups of two up to three animals under controlled conditions (12:12 h light-dark cycle, water and food provided *ad libidum*). After accommodation phase animals were randomly assigned in one of the six following experimental groups: 1. sham-operated (sham); 2. decompressive craniectomy alone (DC); 3. closed head injury alone (CHI); 4. CHI followed by administration of acetazolamide after 2 h (CHI+ACZ); 5. CHI followed by DC at 1 h post-TBI (CHI+DC); 6. CHI+DC followed by administration of acetazolamide after completion of surgical decompression i.e., 2 h post-trauma (CHI+DC+ACZ) (*n* = 8 animals suitable for final analysis in each group).

For groups 3, 4, 5, and 6, experimental TBI was induced using a weight drop device (40). Briefly, the animals were subjected to surgical procedure under isoflurane anesthesia and in body-temperature-controlled environment. After skull exposition, the head was placed on the base of the weight drop device (Laboratory Tools Workshop, Department of Pharmacology, School of Pharmacy, The Hebrew University of Jerusalem, Israel). A 75 g weight was dropped from the height of 30 cm on a silicone cone resting on the exposed skull, resulting in injury to the left hemisphere. For groups 1 and 2 (sham and decompressive craniectomy alone), the same procedure was performed without weight dropping. In the CHI+DC and CHI+DC+ACZ groups, an unilateral DC was performed 1 h after trauma as described previously (31, 41). In brief, a bone flap was created in the parietal and temporal bone using a microsurgical high-speed drill. The temporal bone was then removed down to the skull base and dura was opened above occipital lobe. In DC group, the same procedure was performed on non-traumatized head 1 h following sham injury.

Two hours after CHI application, in groups CHI+ACZ and CHI+DC+ACZ, acetazolamide was administered intraperitoneally (Diamox® parenteral, Mercury Pharmaceuticals Ltd.; 40 mg/kg body weight, diluted in 0.9% NaCl as 2 mg/mL solution = volume of ~ 0.8 mL i.p.). Here the ACZ dosis described in previous mouse experiments dealing with ACZ effects on brain function has been adapted (42). The remaining experimental groups received body-weight adapted volume of sterile 0.9% NaCl solution (~0.8 mL vehicle volume i.p.) as placebo treatment.

After 3 h, temperature probe was removed and anesthesia was withdrawn. Animals were put back into their cages and allowed to recover in an environment with controlled room temperature and subsequently returned to the animal facility.

### Neurological Assessment

The functional status of the animals was evaluated by an observer who was blinded to animal treatment, at 1d and 3d after trauma, before MRI investigations were performed.

A 10-point version of the Neurological Severity Score (NSS) scale was adapted (43) to assess the presence of certain reflexes and the ability to perform motor and behavioral tasks such as beam walking, beam balance, and spontaneous locomotion. Animals are awarded one point for failure to perform a task.

For assessment of balancing skills the modified Beam Balance Score (BBS) with three balancing attempt was used (44). In this test animals are awarded from 0 (good performance) to 5 points (not attempting to balance) and the mean of three attempts was used for further analysis. The total balancing time (maximal 60 s each attempt = maximal total time of 180 s) was analyzed separately.

### Magnetic Resonance Imaging

Serial MRI imaging was performed 1d and 3d after CHI or sham treatment. For MRI, the animals were re-anesthetized with isoflurane and monitored, as described previously (41). MR images were acquired using a system developed for rodent imaging, with a static magnetic field strength of 9.4 T (Bruker BioSpec Avance III 9.4/20). For assessment of tissue damage and edema formation, T1 weighted Multi Slice Multi Echo (MSME), T2 weighted Rapid Acquisition Relaxation Enhanced (RARE) and single shot diffusion weighted echo planar MRI sequences (DWI) were employed (see Table 1), with scan geometry covering at least the entire damaged area.

**Table 1:** Overview of the MRI scan parameter used in the study.

**Imaging modality**	**Sequence**	**Matrix**	**Field of view (cm)**	**TR/TE (ms)**	**NA**	**Slices**	**ST (mm)**	**ID (mm)**	***B* value (s^**2**^ mm^**−1**^)**
T1 weighted	MSME	234 × 200	1.76 × 1.50	1000/10	4	23	0.75	0.0	–
T2 weighted	RARE	234 × 200	1.76 × 1.50	2523/30	8	23	0.75	0.0	–
DWI	EPI	192 × 192	1.92 × 1.92	2000/18.2	1	7	0.75	0.0	6.45/786.74/789.19/789.19^a^

a Effective B values for nominal setting of diffusion weighting of 1000 s mm^−2^ in x, y, and z directions.

*MSME, multi slice multi echo; RARE, rapid acquisition relaxation enhanced; DWI, diffusion weighted imaging; EPI, echo planar imaging; TR, repetition time; TE, echo time; NA, number of acquisitions; ST, slice thickness; ID, interslice distance*.

Brain edema was identified in apparent diffusion coefficient (ADC) maps calculated from the DWI data, and matching Regions of Interest (ROI) were manually created with the Paravision 5.1 ROI tool. Total volumina of the different lesion types (possible cytotoxic edema defined as hypointense areas, possible vasogenic edema defined as hyperintense signal) were calculated from ROI sizes and slice thickness, in Microsoft Excel 2003® for Windows XP®.

### Histological Analysis

In selected groups of animals (CHI+ACZ and CHI+DC+ACZ) additional histopathological analysis of trauma / treatment effects was performed 3d post injury (see also Data Sheet 1 in Supplementary Materials for detailed description of the methods used and for microphotographs).

### Statistical Analysis

Values of mean oxygen flow and mean isoflurane concentration used during surgery and mean head and mean core temperature were expressed as mean ± SEM and analyzed using one-way ANOVA followed by a *post-hoc t*-test with Bonferroni correction for individual comparisons.

For both post injury assessment time points (1d and 3d), neurological impairment according to NSS and BBS, balancing time in BBS test (BBT) as well as volume of both forms of edema (cytotoxic and vasogenic) and total edema volume calculated from MRI scans were expressed as mean ± SEM. Weight of experimental animals (assessed before surgery as well as 24 and 72 h after sham/injury) and change in body weight (Δ weight, as assessed by subtraction of values of two consecutive weightings) were assessed and expressed as mean ± SEM.

In order to assure the Gaussian distribution character of parametric variables (Δ weight, BBT and edema volume) Shapiro-Wilk test retrieving *p* value as validation of normality for single group was performed. For the values with non-Gaussian distribution, as well as for the non-parametric values (NSS and BBS), the rank-based Kruskal-Wallis test followed by Dunn's multiple comparison *post-hoc* test was applied. For the parametric values with Gaussian distribution statistical comparison was performed using one-way ANOVA with Bonferroni *post-hoc* test. Analysis was performed adjusting *p* values for multiple comparisons (all six groups, seven relevant comparison pairs) as well as within sets of groups (categorized according to decompression status i.e., the subset of animals without craniectomy: sham, CHI and CHI+ACZ and the subset of animals with craniectomy: DC, CHI+DC, CHI+DC+ACZ; three relevant comparison pairs for each set). Sham group served as reference in subset of animals without craniectomy, while DC group served as reference in subset of animals with craniectomy (31). Comparisons with adjusted *p* values that were significant in grouped analysis (Kruskal-Wallis or one-way ANOVA) were verified by comparing pairs of treatment groups (e.g., CHI vs. CHI+ACZ) at the corresponding time point using Mann-Whitney or unpaired *t*-test, respectively, in order to obtain *p* value for each relevant pair of comparison.

Secondary analysis assessed, whether the impairment score, edema volume and Δ weight changed significantly over time within single treatment groups. Here, Friedman's two-way analysis of the variance by ranks was used, treating each mouse as a block and time as a factor.

To analyze the correlation between the size of structural damage (volume of edema) and the neurological impairment (NSS, BBS, BBT) and weight loss (as indirect indicator of the diuretic effect) the assessed data from single groups were pooled according to time point of assessment. In order to extract potential impact on ACZ on relationship between structural and functional sings of injury, groups of TBI animal with placebo treatment (CHI and CHI+DC) and with ACZ treatment (CHI+ACZ and CHI+DC+ACZ) were pooled separately. To avoid outliner's effect, both reference groups (sham and DC with zero-value or near-zero values) have been excluded from this part of analysis. Thereafter the Pearson correlation coefficient analysis and a subsequent linear regression analysis method were performed.

For all parts of assessment (analysis of variance, correlation analysis), significance was set at *p* < 0.05 and statistical software GraphPad Prism version 5.00 for Windows, GraphPad Software, San Diego California USA, www.graphpad.com as well as IBM SPSS Statistics for Windows, Version 22.0 IBM Corp. Released 2013. Armonk, NY: IBM Corp. was used.

## Results

### Perioperative Management

The analysis of the mean core/head temperature, mean oxygen flow and mean isoflurane concentration used during anesthesia showed no differences between the subgroups.

### Mortality

The use of prolonged anesthesia resulted in mortality of 20% among sham animals. Half of them (50%) died before pharmacologic treatment (placebo or ACZ administration, arbitrary defining the early mortality in this study). In turn, craniectomy itself (DC group) was burdened with total mortality of 37% (early mortality of 14%). Following CHI, both trauma and trauma + craniectomy treatment resulted in 50% mortality. The highest mortality scores were recorded among ACZ-treated animals (72% for CHI+ACZ group and 76% for CHI+DC+ACZ group). Notably, early mortality fraction reached 50% in both groups treated with posttraumatic craniectomy (CHI+DC and CHI+DC+ACZ), while in animals without decompression the death occurred rather in prolonged course (early mortality of 25% in CHI group) or, more precisely, after ACZ administration (early mortality of 29% among CHI+ACZ animals).

### Weight of Animals

Animal body weights did not differ initially between the groups (*p* > 0.05, ns).

**Figure 1:**
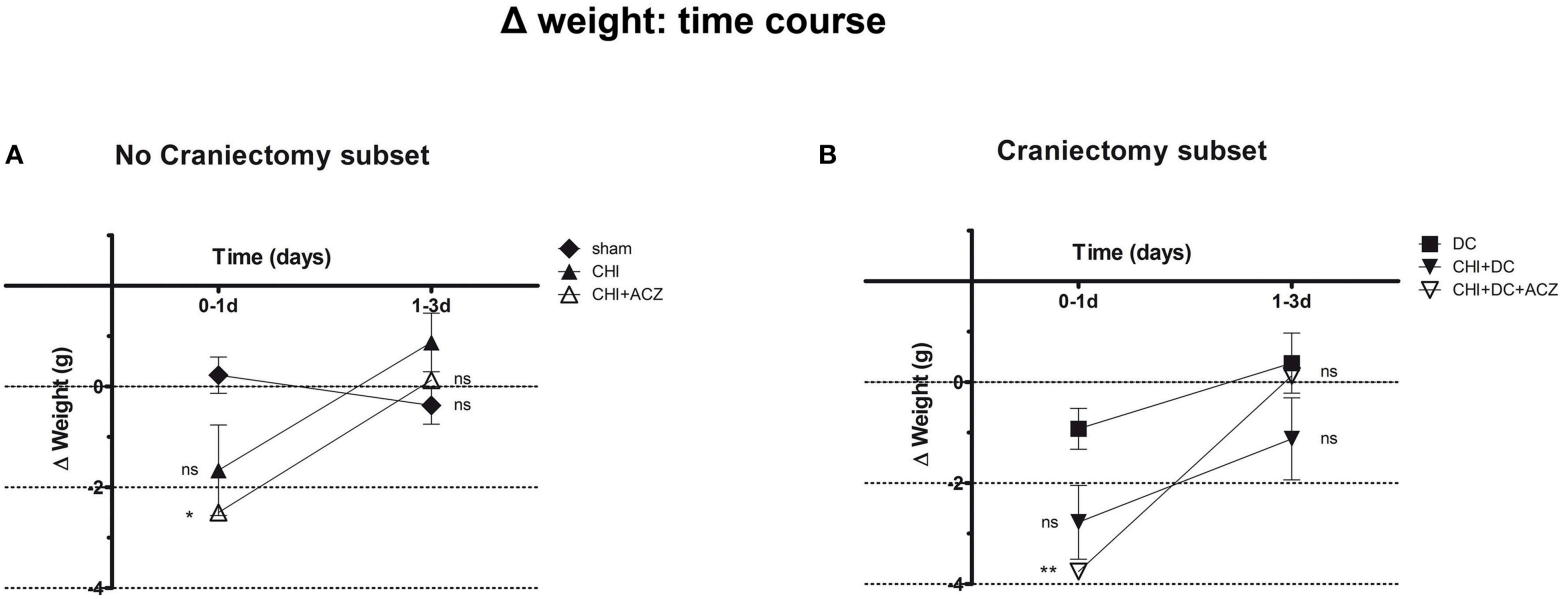
Graph demonstrating detailed analysis of body weight changes (Δ weight) in experimental animals during early time course (up to 72 h = 3d) after trauma/sham injury. **(A)** In subset of animals without craniectomy, the most dramatic weight loss during first 24 h after injury could be demonstrated in animals treated by acetazolamide administration (CHI+ACZ vs. sham, 1d: ^*^p < 0.05). Later on (from 1d to 3d of posttraumatic recovery), no significant differences between the treatment groups could be documented (3d: *p* > 0.05, ns for all group comparison within the subset). **(B)** The most dramatic weight loss during first 24 h after injury among craniectomy subset (and among all groups) could be demonstrated in animals treated by trauma, decompression and acetazolamide administration (CHI+DC+ACZ vs. DC, 1d: ^**^p > 0.01). Nonetheless, also in these animals, no further significant differences in weight changes could be documented from 1d to 3d post injury (3d: *p* > 0.05, ns for all pairs of groups). CHI, closed head injury; DC, decompressive craniectomy; ACZ, acetazolamide.

Detailed analysis of weight changes in postoperative course (Δ weight) revealed significant differences during the first 24 h postoperative hours following surgery (Figure 1), but not thereafter i.e., at 3d post injury). Both ACZ treated groups showed a more profound weight loss when compared to sham treated and DC animals, respectively (Δ weight at 1d: sham 0.23 ± 0.36 g vs. CHI+ACZ−2.5 ± 0.85 g; *p* < 0.05; DC−0.93 ± 0.4 g vs. CHI+DC+ACZ−3.75 ± 0.59 g; *p* < 0.01).

Two-way analysis revealed a significant effect of time on the weight changes (*p* = 0.001 for time effect).

### Functional Outcome

**Figure 2:**
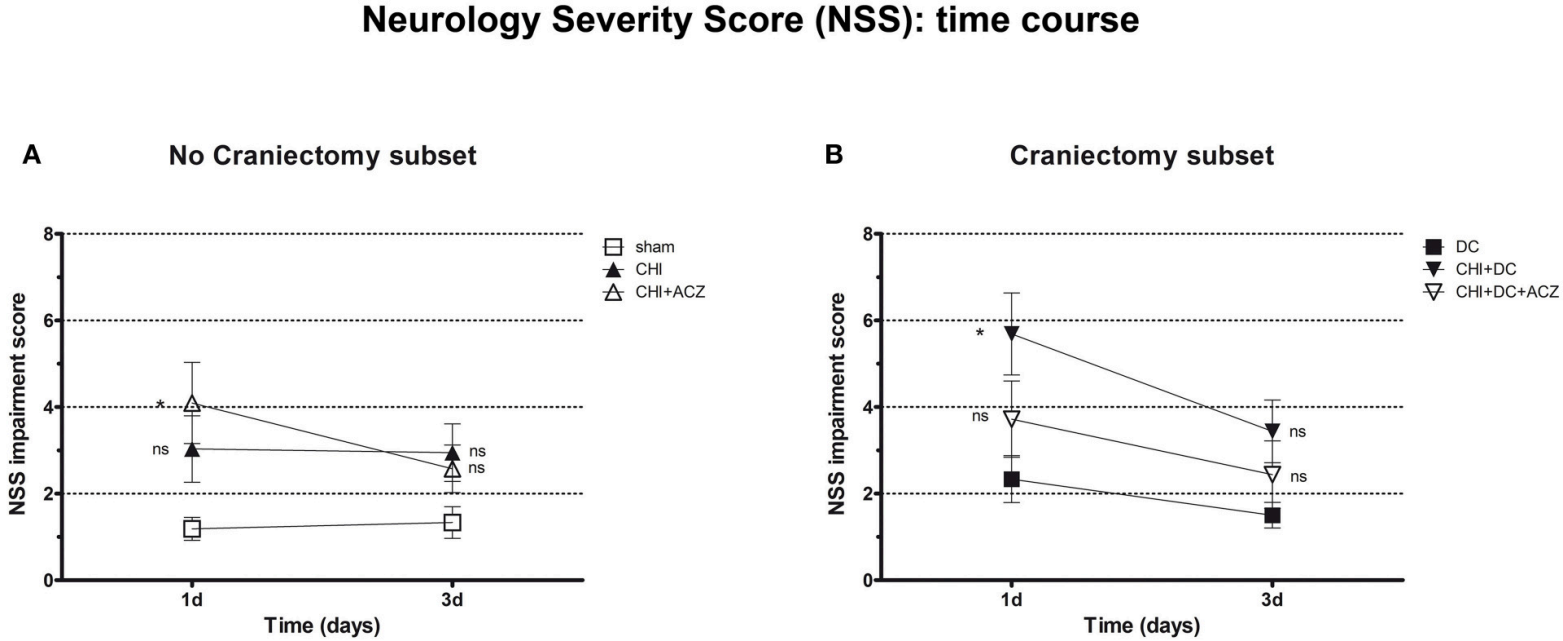
Graph showing neurological impairment during early time course (up to 72 h = 3d) after trauma/sham injury according to neurological severity score (NSS). **(A)** In subset of animals without craniectomy, animals treated with ACZ administration demonstrated the significant deterioration compared to sham group (CHI+ACZ vs. sham, 1d: ^*^p < 0.05). Due to functional recovery, no significant difference between groups could be recorded at 3d post injury (3d: *p* > 0.05, ns for all group comparisons within the subset). **(B)** In turn, among groups treated with craniectomy, the profound functional deterioration could be demonstrated at 1d only in animals without ACZ administration, as compared to reference group (CHI+DC vs. DC, 1d: ^*^p < 0.05). Also here, improvement in early outcome at 3d could be documented (3d: *p* > 0.05, ns for all group comparisons within the subset). CHI, closed head injury; DC, decompressive craniectomy; ACZ, acetazolamide.

According to NSS, CHI animals treated with ACZ presented a significantly poorer performance 1d after injury, when compared to sham animals (NSS at 1d: CHI+ACZ 4.09 ± 0.94 vs. sham 1.19 ± 0.27; *p* < 0.01). However, among animals with DC, those subjected to trauma and craniectomy (CHI+DC) performed the poorest 1d after injury (NSS at 1d: CHI+DC 5.69 ± 0.95 vs. DC 2.33 ± 0.54; *p* = 0.02). Interestingly, this effect of DC was attenuated by administration of ACZ (NSS at 1d: CHI+DC+ACZ 3.72 ± 0.88 vs. DC 2.33 ± 0.54; *p* = 0.25). During further recovery (time point 3d), significant differences between the groups could not be demonstrated (NSS at 3d: *p* > 0.05 for all combinations in the both subgroups) (Figure 2).

**Figure 3:**
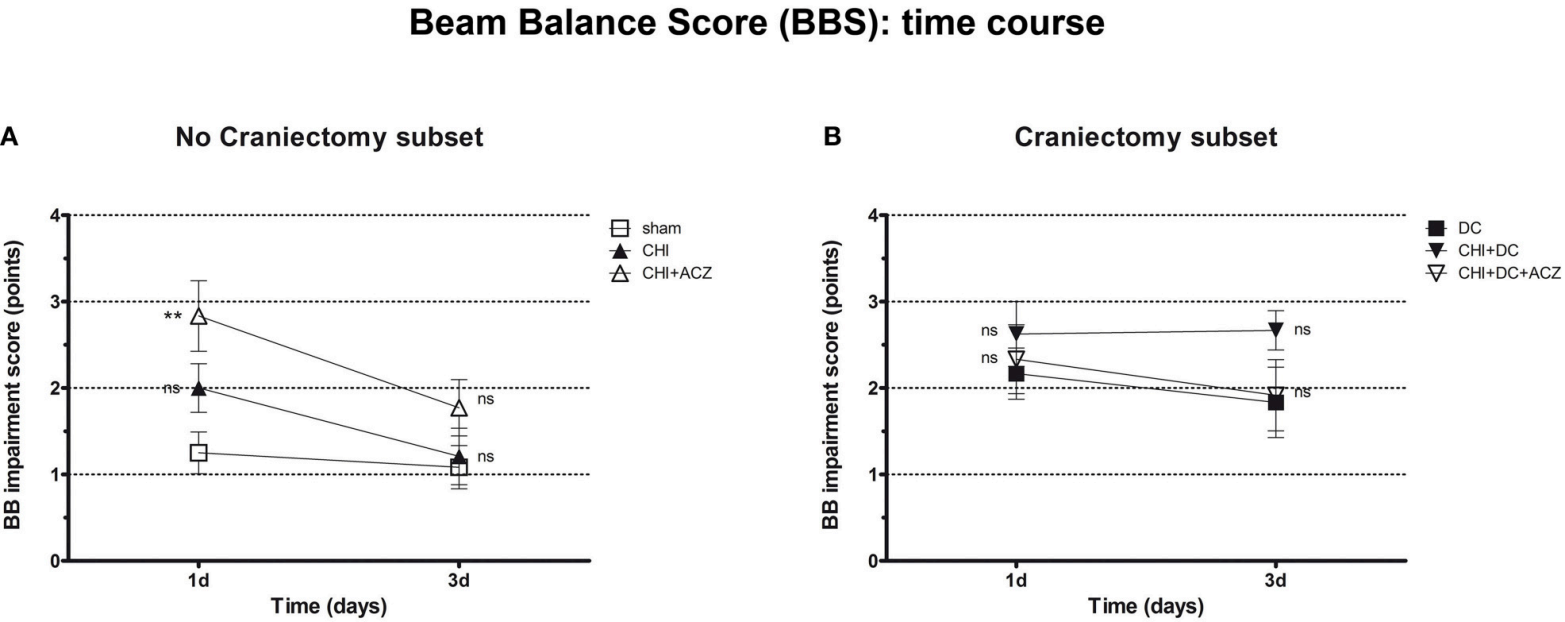
Graph showing neurological impairment during early time course (up to 72 h = 3d) after trauma/sham injury according to Beam Balance Score (BBS). **(A)** Similar to NSS results, animals treated with ACZ displayed the worst performance at 1d among non-craniectomy subset (and among all groups) (CHI+ACZ vs. sham, 1d: ^**^p < 0.01). Again, functional recovery led to reduction of functional impairment at 3d (3d: *p* > 0.05, ns for all group comparisons within the subset). **(B)** In contrast, among craniectomy subset, no significant differences in BBS could be documented at both time points analyzed (1d and 3d: *p* > 0.05, ns for all group comparisons within the subset). CHI, closed head injury; DC, decompressive craniectomy; ACZ, acetazolamide.

Results of BBS showed a comparable pattern of functional impairment both at 1d and 3d post injury. Again, animals subjected to both CHI and ACZ administration presented with significantly poorer performance comparing than sham littermates at 1d (BBS at 1d: CHI+ACZ 2.83 ± 0.41 vs. sham 1.25 ± 0.24; *p* = 0.01). At the same time point, differences in BBS were not significant for animals treated with DC and ACZ compared to DC alone (BBS at 1d: CHI+DC+ACZ 2.33 ± 0.40 vs. DC 2.17 ± 0.30; *p* = 0.71) Contrary to NSS, performance of the CHI+DC groups showed no significant difference to its reference (BBS at 1d: CHI+DC 2.63 ± 0.38 vs. DC 2.17 ± 0.30; *p* = 0.66). In the further course of the experiment, significant differences among the subgroups were diminished by recovery of the animals at 3d post injury (BBS at 3d: *p* > 0.05 for all combinations in both subgroups) (Figure 3).

**Figure 4:**
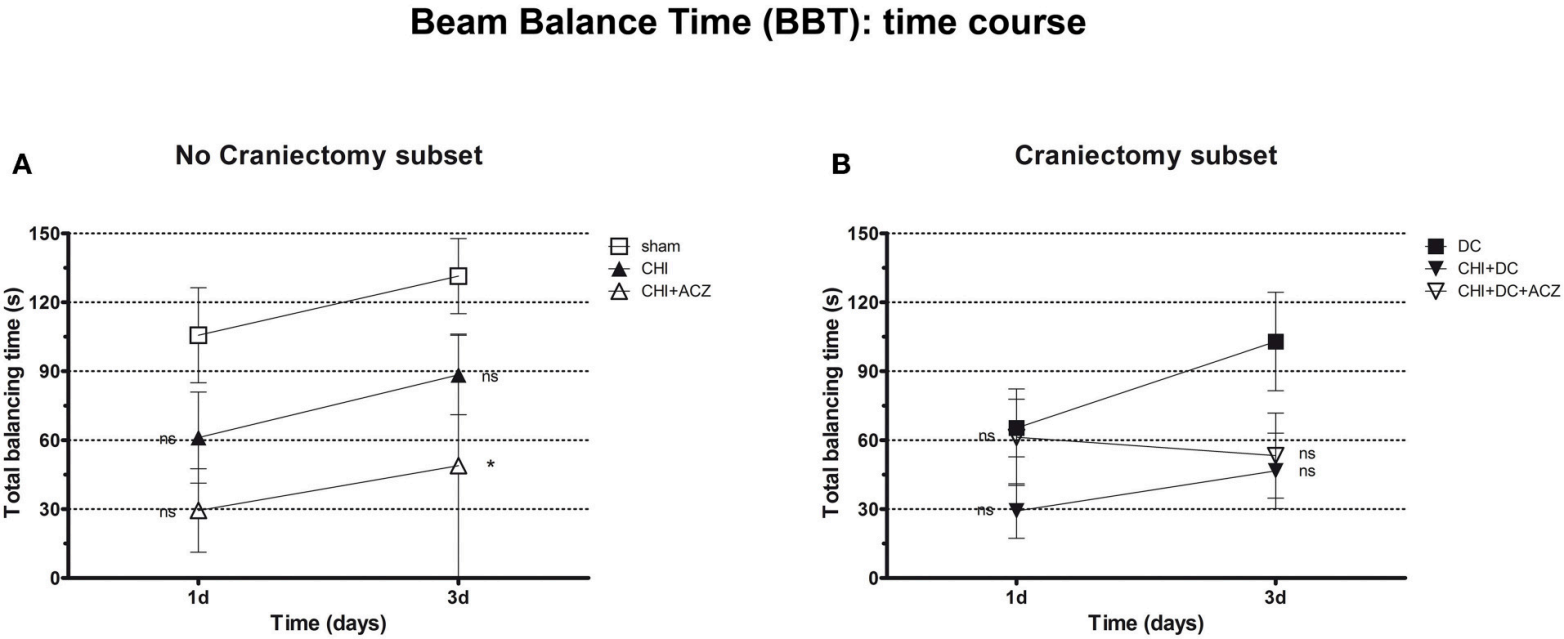
Graph presenting neurological impairment during early time course (up to 72 h = 3d) after trauma/sham injury according to total Beam Balancing Time (BBT) in beam balance test. **(A)** In this analysis, animals treated with ACZ administration demonstrated the significant impairment (shortening of balancing time) not before 3d post trauma, as compared to sham group (CHI+ACZ vs. sham, 3d: ^*^p < 0.05). Increasing total balancing times represent the overall tendency of neurological recovery across the time. **(B)** In turn, among groups treated with craniectomy, no significant differences in balancing time could be stated (similar to impairment score in BBS). In graphic presentation, the recovery effect seems to be affected in animals with additional ACZ treatment (CHI+DC+ACZ), however, without reaching the level of significance in variance analysis (1d and 3d: *p* > 0.05, ns for all group comparisons within the subset). CHI, closed head injury; DC, decompressive craniectomy; ACZ, acetazolamide.

Analysis of total balancing time yielded different results: While both in craniectomy subset and in no-craniectomy subset no significant differences could be documented 1d after trauma (BBT at 1d: *p* > 0.05 for all combinations in both of subgroups), at 3d animals exposed to ACZ treatment without decompression demonstrated significant reduction in balancing time as compared to sham treated mice (BBT at 3d: CHI+ACZ 48.88 ± 20.24 s vs. sham 131.4 ± 16.36 s; *p* = 0.014). Notably, this negative effect was not seen in animals, in which ACZ administration was preceded by surgical decompression (BBT at 3d: CHI+DC+ACZ 53.25 ± 18.53 s vs. DC 102.9 ± 21.41 s; *p* = 0.13) (Figure 4).

Two-way analysis of neurological function revealed a significant effect of time on functional impairment in NSS and balancing time assessment (NSS: Friedman's two-way analysis of variance by ranks: *p* = 0.01, ^**^ for time effect; BBT: Friedman's two-way analysis of variance by ranks for time effect: *p* = 0.025, ^*^ for time effect) but not for BBS assessment (BBS: Friedman's two-way analysis of variance by ranks for time effect: *p* > 0.05, ns for time effect).

### Radiological Assessment of Edema

**Figure 5:**
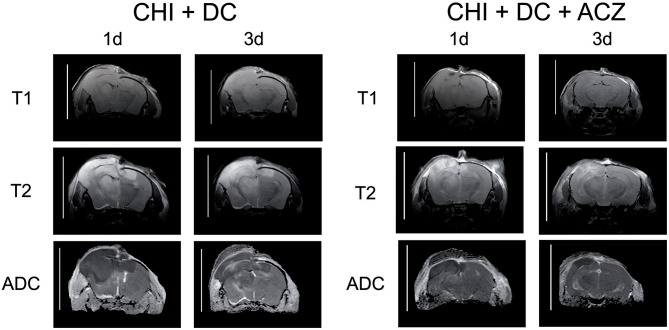
Illustrating the difference in brain edema course, observed between the animals treated with acetazolamide after trauma/decompression and between placebo-treated group. MRI-scans (T1 sequence, T2 sequence, and apparent diffusion coefficient (ADC) map) were obtained from animals subjected to closed head injury followed by decompressive craniectomy (CHI+DC) without acetazolamide (ACZ) administration and from animals with additional i.p. administration of ACZ (CHI+DC+ACZ). The scanning was performed 1d and 3d postinjury. The animals with craniectomy applied after CHI presented significant amount of brain edema. Already 1d postinjury, a part of edema displayed vasogenic properties, as expressed by hyperintensity in ADC map sequence. In contrast, in animals with additional ACZ treatment both cytotoxic (hypointense) and, strikingly, vasogenic (hyperintense) edema was less manifest at both analyzed time points. The scans were obtained in representative animals using 9.4 T MRI scanner; bar = 10 mm.

ADC maps created from DWI experiments demonstrated edematous areas as hypointense at 1d after injury. Hyperintense areas were detected in damaged brain only 3d post injury and were largest in animals subjected to both trauma and craniectomy (Figure 5). Animals that received ACZ treatment presented with only scarce areas of ADC-hyperintense edema (Figure 5). In all groups subjected to trauma, hypointense (cytotoxic) edema presenting with lower ADC dominated the small portions of hyperintense (vasogenic) edema with higher ADC. Accordingly, calculation of total edema represents mainly the differences in cytotoxic-like edema extent.

**Figure 6:**
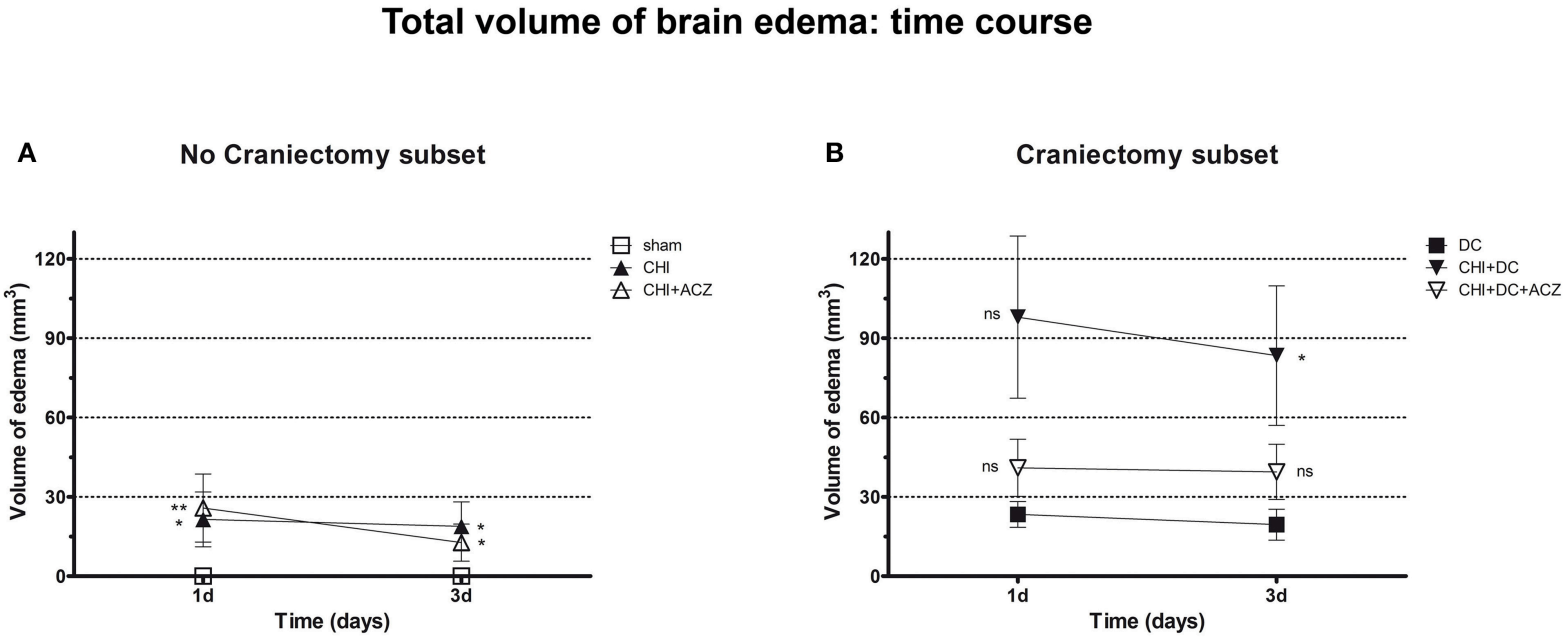
Graph demonstrating results of volumetric brain edema analysis after trauma/sham injury as visible in apparent diffusion coefficient (ADC) maps. **(A)** Quantitative volumetric analysis revealed significantly increased amount of cerebral edema at 1d and 3d postinjury, both in animals treated with placebo (CHI vs. sham, 1d and 3d: ^*^p < 0.05) and with acetazolamide (CHI+ACZ vs. sham, 1d: ^**^p > 0.01; 3d: ^*^p < 0.05). Note, that the sham animals (serving as reference group in no-craniectomy subset) displayed no discernible brain edema at all. **(B)** In contrast, among groups treated with craniectomy the animals without ACZ administration demonstrated the most severe cerebral edema, becoming significant at 3d after trauma, as compared to reference littermates (CHI+DC vs. DC, 3d: ^*^p < 0.05). Note, that this deleterious effect of trauma/decompression was not discernible in animals with additional acetazolamide administration (CHI+DC+ACZ, *p* > 0.05, ns for both time points). CHI, closed head injury; DC, decompressive craniectomy; ACZ, acetazolamide.

Analyzing subset of animals without craniectomy, significant differences could be demonstrated between animals with trauma and sham treated group (total edema 1d: CHI 21.47 ± 10.35 mm^3^ vs. sham 0.0 ± 0.0 mm^3^; *p* < 0.05; total edema 3d: CHI 18.81 ± 9.28 mm^3^ vs. sham 0.0 ± 0.0 mm^3^; *p* < 0.05) and for animals with ACZ treatment and sham mice (total edema 1d: CHI+ACZ 25.75 ± 12.85 mm^3^ vs. sham 0.0 ± 0.0 mm^3^; *p* < 0.01; total edema 3d: CHI+ACZ 11.11 ± 6.28 mm^3^ vs. sham 0.0 ± 0.0 mm^3^; *p* < 0.05, ns) (Figure 6A). Remarkably, in subset of animals without craniectomy, sham animals did not demonstrate any discernible edema (total edema at both 1d and 3d: sham 0.0 ± 0.0 mm^3^).

About DC subset: Total brain edema volume was biggest in CHI+DC mice across all groups, but differed significantly from the DC group only 3d after trauma induction (total edema 1d: CHI+DC 97.96 ± 30.66 vs. DC 23.34 ± 4.89 mm^3^; *p* = 0.10; total edema 3d: CHI+DC 83.38 ± 26.38 vs. DC 19.49 ± 5.85 mm^3^; *p* = 0.03). No significant differences were found for animals with additional administration of ACZ (total edema 1d: CHI+DC+ACZ 40.96 ± 10.81 mm^3^ vs. DC 23.34 ± 4.89 mm^3^, *p* = 0.28; total edema 3d: CHI+DC+ACZ 39.44 ± 10.35 mm^3^ vs. DC 19.49 ± 5.85 mm^3^, *p* = 0.11) (Figure 6B).

### Correlation of Functional Outcome and Radiological Assessment

**Figure 7:**
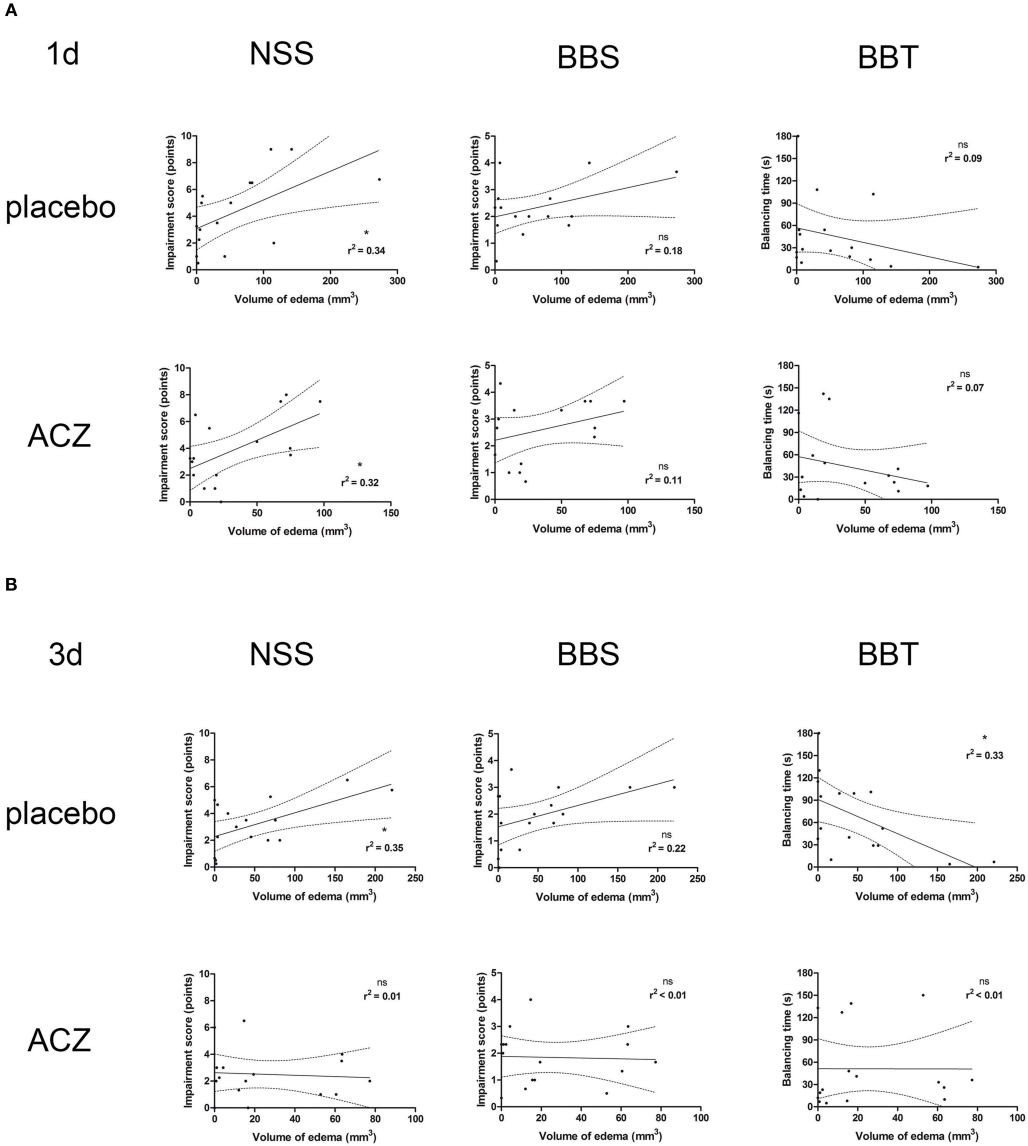
Histogram presenting analysis of correlation between volume of edema and neurological outcome. Only the traumatized animals has been included in this analysis (see Methods and Statistical analysis). The groups of graphs **(A,B)** represent different time points (1d and 3d, respectively). In each group, the upper row displays correlation analysis in placebo-treated animal, while lower row represents results of the same analysis in animals receiving ACZ. The columns are arranging single graphs according to functional variable (NSS, BBS, and BBT, respectively) subjected to linear regression analysis along with total volume of edema. The description of statistical results refers to both probability of true correlation (as expressed by *p*-value; ns for *p* > 0.05 and ^*^p < 0.05) as well as to the strength of the correlation (as expressed by *r*^2^-value, varying between 0 for no correlation and 1 for very strong correlation). **(A)** At 1d after injury, a positive correlation could be demonstrated in both subsets of animals, according to NSS score (for placebo subset: edema vs. NSS: *r*^2^ = 0.34, ^*^p < 0.02; for ACZ subset edema vs. NSS *r*^2^ = 0.32, ^*^p = 0.02). **(B)** Up to 3d post injury, the correlation between volume of edema and neurological impairment (according to NSS and balancing time) was sustained among placebo-treated animals (for placebo subset: (edema vs. NSS *r*^2^ = 0.35, ^*^p < 0.02; edema vs. balancing time *r*^2^ = 0.33; ^*^p = 0.02). In contrast, ACZ-treated animals demonstrated no correlation between neurological function and between the volume of edema at this time point. Note the course of correlation line in lower row (ACZ), almost parallel to x-axis, indicating dissociation of relationship between analyzed variables. ACZ, Acetazolamide; NSS, Neurology Severity Score; BBS, Beam Balance Score; BBT, Beam Balancing Time.

Correlation analysis was performed separately for the subsets of traumatized animals with placebo treatment (CHI and CHI + DC) and with ACZ administration (CHI+ACZ and CHI+DC+ACZ). At 1d post treatment (Figure 7A), volume of edema correlated well with NSS score, but not with BB or BBS, in both placebo (edema vs. NSS: *r*^2^ = 0.34, *p* < 0.02; edema vs. BB *r*^2^ = 0.18, *p* = 0.11, ns; edema vs. balancing time *r*^2^ = 0.09; *p* = 0.25) and in ACZ subset: (edema vs. NSS *r*^2^ = 0.32, *p* = 0.02; edema vs. BB *r*^2^ = 0.11, *p* = 0.21; edema vs. balancing time *r*^2^ = 0.07; *p* = 0.32). At 3d post injury (Figure 7B), NSS and BBT but not BBS showed a significant correlation with edema volume only in placebo treated animals (edema vs. NSS *r*^2^ = 0.35, *p* < 0.02; edema vs. BB *r*^2^ = 0.22, *p* = 0.07; edema vs. balancing time *r*^2^ = 0.33; *p* = 0.02) and not in ACZ subset (edema vs. NSS *r*^2^ = 0.005, *p* = 0.79; edema vs. BB *r*^2^ = 0.002, *p* = 0.88; edema vs. balancing time *r*^2^ = 0.000006; *p* = 0.99).

Correlation analysis between volume of edema and weight loss (as indirect measure of diuretic effect) demonstrated a significant positive correlation between both variables at 1d post injury for animals treated with ACZ (edema vs. weight loss *r*^2^ = 0.54, *p* = 0.0013) but not thereafter (at 3d: edema vs. weight loss *r*^2^ = 0.002, *p* = 0.87). In turn, in animals with placebo treatment no correlation between edema volume and weight loss could be demonstrated at earlier time point (1d: edema vs. weight loss *r*^2^ = 0.25, *p* = 0.0512) while at 3d there was a strong significant correlation between variables (3d: edema vs. weight loss *r*^2^ = 0.49, *p* = 0.0025).

## Discussion

In this study, we have investigated the therapeutic potential of ACZ as adjuvant treatment applied after decompressive craniectomy, when the skull needs to be opened due to life-threatening consequences of severe TBI. While DC under such conditions is regarded a life saving measure (45), additional brain damage and functional impairment due to DC have been suggested in some reports on complications of DC (29) and in one large prospective clinical trial (33). This effect was also demonstrated in our previous experiments mimicking the time course of TBI and subsequent treatment procedures in small laboratory animals (31, 46). This time we could describe two-faced properties of ACZ administration in regard to posttraumatic course with rather positive effect in craniectomized animals and rather deleterious action in animals without previous surgical decompression.

Our current experimental results support the previous findings, showing most profound functional impairment and brain edema after application of both trauma and decompression (31, 46). Reaching beyond the time point of peak in posttraumatic edema and impairment (40, 47) spontaneous recovery of trauma sequelae was observed in all treatment groups. These results are also in line with previous experimental studies on CHI and on DC (40, 47–49) and with clinical studies, presenting early and prolonged recovery after injury and decompression (50–52). We conclude that our elaborate animal model is suitable for translational research, since it reproduces the main features of DC relevant to the clinician, and various sequelae of brain damage and treatment that TBI patients go through are mimicked.

To overcome adverse effects of DC, a concept of supplementary treatment has been proposed by us and has already been successfully tested in animals, showing a mitigation of brain edema formation and of neurological impairment, when combining decompressive craniectomy with focal cooling of traumatized brain areas (46, 53). As an alternative supplementary treatment for DC side effects, in this study we employed the mild diuretic ACZ, since its administration has been shown to provide neuroprotective and antiedematous action (3, 14). However, ACZ increases intracranial blood volume and may therefore, exert negative effects (17, 24, 25).

As demonstrated by our experiments, ACZ administration interfered with the pattern of functional impairment and recovery in CHI, and effects of ACZ treatment were ambiguous. ACZ treatment resulted in increased mortality of animals with severe TBI and CHI + DC. We attribute this negative effect to the enhanced diuresis following ACZ application, since both animal groups receiving ACZ presented with lower body weight than their specific controls.

However, this diuretic effect might be as well a prerequisite for the positive effect of reduction of brain edema observed in this study after ACZ administration. In animals that survived CHI, ACZ treatment resulted in an attenuation of the brain edema increase present in craniectomized animals. ACZ also ameliorated functional impairment caused by trauma and surgical manipulation. However, the exact pharmacological mechanisms leading to these partially beneficial and partially detrimental results have not been tackled by this investigation. Therefore, it remains unclear, whether beneficial effects of ACZ arise from its central or peripheral effects on water homeostasis, on blood flow or pH.

Moderate primary diuresis as a result of ACZ administration (10, 11) might be in this context an effective underlying mechanism, since it might be followed by secondary brain dehydration and limitation of brain edema (12, 13).

Perhaps the most appealing hypothesis is that ACZ effects in our experimental model are mediated via water channel proteins. As recently described, ACZ is able to limit water permeability of cell membranes by blocking the function of aquaporin-4 (AQP4) (3, 4, 6, 54). Furthermore, it is regarded as the main water channel protein responsible for brain edema formation following trauma to the brain (55–59). However, its role is ambiguous and strongly depends on the specific pathophysiological basis of brain edema (55, 58–61). AQP4 seems to play a crucial role in formation of cytotoxic edema. Increase in AQP-mediated water permeability leads to more extensive cellular swelling. Accordingly, in animal experiments employing pharmacological inhibition as well as AQP4 gene knockout result in reduction of cytotoxic edema (62–64). This hypothesis is also supported from a previous study employing the animal model used in this investigation, where a significant correlation between edema volume and AQP4 expression level could be demonstrated (41). However, for the more conclusive description of ACZ action on AQP4 role after DC, additional experimental series with more elaborated biochemical analyses will be required.

In formation of vasogenic edema, the pathophysiology of AQP4 is reversed. Here, increase of activity promotes water resolution. Thus, any intervention impairing AQP4 function may exacerbate this type of swelling (65–67). Vasogenic edema, caused by increased vascular permeability is an important injury component in CHI but is detected in the later course during recovery (68, 69). In accordance, in our experiments hyperintense areas in ADC maps indicating vasogenic edema can only be found 3d after trauma induction. Moreover, the dissociation of relationship between edema volume and degree of neurological impairment, as demonstrated by correlation analysis, suggest a change of edema character caused by acetazolamide administration. Also, our findings in CHI+DC treated animals fit with the current doctrine that formation of vasogenic edema is facilitated by a shift of water from intravascular to extracellular space driven by an intravascular/intracranial pressure gradient (70–72). In this context, the sparsity of vasogenic edema (estimated from ADC maps) in CHI+DC+ACZ treated mice was an unexpected finding: Since ACZ increases cerebral blood flow and intracranial blood volume by vasodilation (1, 18), increased permeation of water into brain parenchyma was anticipated. However, cerebral vasodilatation by ACZ may also be related to a decrease in cerebral perfusion pressure (73) including microvascular pressure. This in turn may result in reduced transcapillary filtration and in ameliorated vasogenic swelling.

Edema reduction by ACZ may also be connected to its negative effect on CSF production. Disturbance in CSF turnover has been reported at an early time point during disease progression following trauma, and CSF water may passively enter periventricular white matter by diffusion (74, 75). Possibly, this phenomenon is responsible for part of hyperintense MRI changes seen in ADC maps. ACZ is a potent inhibitor of CSF secretion (76–78) and this property has been used in treatment of benign intracranial hypertension (79) as well as some forms of hydrocephalus (80). Interestingly, CSF formation seems to be mediated by AQP1 (81), another water channel that can be inhibited by ACZ.

The positive effects of ACZ administration in animals treated with DC observed in this study argue strongly for further investigation of the underlying pathophysiological mechanisms. In order to get a more detailed insight into the process of edema formation, further studies need to be performed. Such investigations may include determination of physiologic parameters like intracerebral and blood pressure and potentially compare pharmacological intervention with ACZ and more selective AQP antagonists. Such substances have already been identified and investigated in other experimental models, including studies on healthy animals (81), cancer cells (82), and stroke (83).

In our experimental study, the selected treatment combination (trauma ± craniectomy ± ACZ) showed an influence on animal recovery. Adding both ACZ and DC to the treatment regime of CHI animals was particularly beneficial for both edema reduction and performance in neurologic tests, since profound impairment as seen in the group of animals without ACZ (CHI + DC) 1d post injury could be prevented. This positive effect of ACZ administration was not demonstrated in animals not receiving DC, thus confirming our presumption, that the beneficial action of ACZ may be masked by negative aspects of vasodilation in the constricted cranial vault. However, the effects of single ACZ administration were less prominent at 3d post trauma. This may be attributed to the limited effectivity of ACZ treatment, since only a single dose was applied (1, 3). Furthermore, differences between treatment and sham groups become less manifest due to spontaneous neurological recovery (40), as supported by the significant time effect demonstrated by two-way analysis.

In this study, we have for the first time demonstrated the beneficial use of ACZ, in a treatment regime for acute severe TBI. Improvement of the posttraumatic course of animals could be achieved by only a single dose of the substance. While ACZ has been widely used in neurosurgical practice (15–22, 80, 84, 85), translating our treatment regime into clinical practice is hampered by a couple of shortcomings that need to be addressed in follow-up studies. First, while the CHI was deliberately chosen to perform DC on the not previously trephined skull, and to demonstrate a well-delineated injury epicenter by MRI (31, 49), this model does not necessarily demonstrates all the features of severe head trauma as seen in clinical routine. In particular, acceleration-deceleration effects, resulting in traumatic axonal injury are less prominent in CHI than in other experimental TBI models (86). Second, the experimental protocol had to be designed as a feasibility study with a low number of animals that could be investigated, therefore limiting statistical power, long term follow up and ACZ dose. Third, for the sake of comparability of results, we have retained the setting of our original CHI and decompressive craniectomy description (31). Thus, only a limited set of parameters has been investigated, without adding more complex invasive measurement methods like ICP or intra-arterial blood pressure. Nevertheless, important data about the impact of ACZ on posttraumatic events have been gathered. In particular, the primary presumption, that ACZ would further increase vasogenic edema formation could be rebutted. Our investigation creates a solid fundament for subsequent experiments aimed at revealing the full potential of ACZ in treatment of acute TBI.

In our experimental study single administration of ACZ during acute post traumatic phase of closed head injury attenuates brain swelling and alleviates functional impairment resulting from mechanical trauma and surgical manipulation, if applied after decompressive craniectomy, while the underlying mechanisms remain unclear. AQP1 and AQP4 are candidate targets of ACZ and may be exploitable more selectively in the future. Decompressive craniectomy (if performed for any reason during posttraumatic course) potentially counteracts negative effects of ACZ administration, likewise preventing ICP raise related to ACZ-caused vasodilation. Nonetheless, further exploration of the complex biophysical and molecular interactions following craniectomy is recommended, before implementing ACZ into clinical TBI trials.

## Data Availability

The datasets generated for this study are available on request to the corresponding author.

## Author Contributions

JS, VH, AEM, KS, and JO contributed conception and design of the study. JS, VH, and EK conducted the animal experiments. AM, VH, EK, and LFA performed the radiological assessment. JS, VH, EK, and LFA performed the histopathological analysis. JS and AM performed the statistical analysis. JS wrote the first draft of the manuscript. AM and VH wrote sections of the manuscript. All authors contributed to manuscript revision, read, and approved the submitted version.

### Conflict of Interest Statement

The authors declare that the research was conducted in the absence of any commercial or financial relationships that could be construed as a potential conflict of interest.

## Abbreviations

ACZ: acetazolamide
ADC: apparent diffusion coefficient
AQP4: aquaporin 4
BBS: beam balance score
BBT: beam balance time
CCI: controlled cortical impact
CHI: closed head injury
CSF: cerebrospinal fluid
DC: decompressive craniectomy
DWI: diffusion weighted imaging
FPI: fluid percussion injury
ICP: intracranial pressure
MRI: magnetic resonance imaging
MSME: Multi Slice Multi Echo
NSS: neurological severity score
RARE: Rapid Aquisition Relaxation Enhanced
ROI: region of interest
TBI: traumatic brain injury.
