# Supplementary material for: Brain Edema Formation and Functional Outcome After Surgical Decompression in Murine Closed Head Injury Are Modulated by Acetazolamide Administration
Source: Front Neurol. 2019 Mar 26;10:273. doi: 10.3389/fneur.2019.00273 (PMC6443632; doi:10.3389/fneur.2019.00273)
Supplement: Supplementary file 1 [file Data_Sheet_1.docx]

**Brain edema formation and functional outcome after surgical decompression in murine closed head injury are modulated by acetazolamide administration**

*Jacek Szczygielski, Vanessa Hubertus, Eduard Kruchten, Andreas Müller,*

*Lisa F. Albrecht, Angelika E. Mautes, Karsten Schwerdtfeger and Joachim Oertel*

SUPPLEMENTARY DATA

**Supplementary Materials and Methods (Histology and Immunohistochemistry)**

Animals subjected to acetazolamide treatment were sacrificed using transcardial perfusion with buffered formaldehyde solution; the brains were removed and fixed in the same solution for 7 days. The brains were paraffin embedded and serial coronal sections of the brains (5 µm) were made, presenting region of interest (ROI) i.e. the coronal slices displaying hippocampal areas CA1 and CA3. Thereafter, classical histopathological staining (hematoxylin & eosin (HE) and Nissl) were performed. Further slices were immunostained with anti-GFAP and anti-AQP4 antibodies according to following procedure: Coronal sections were deparaffinized and rehydrated. Antigen retrieval was performed by boiling sections in 10 mM citrate buffer (pH 6.0) for 20 min. After cooling for 20 min and washing with TRIS puffer, sections were incubated with 0.3% H_2_O_2_ in methanol for 20 min and washed with TRIS, thereafter intrinsic binding sites were blocked using 5% goat serum plus biotin/avidin blocking kit (Vector Laboratories; SP-2001), and incubated with rabbit polyclonal AQP4 antibody with mouse reactivity (Santa Cruz Biotechnology; sc-20812, dilution 1:100) or rabbit polyclonal GFAP antibody (Abcam, ab-16977, dilution 1:100) at 4 °C overnight. After washing with TRIS puffer, sections were incubated with biotinylated goat anti-rabbit antibody (Vectastain Elite ABC HRP Kit (Peroxidase Rabbit IgG) Vector Laboratories; PK-6101) at room temperature for 60 min and thereafter washed with TRIS puffer for 10 min. Following, sections were incubated with an avidin-biotin-peroxidase system (Vectastain Elite ABC HRP Kit (Peroxidase Rabbit IgG) Vector Laboratories; PK-6101) for 20 min and washed with TRIS puffer for 10 min. Thereafter slices were stained using chromogen kit (Vector VIP Peroxidase HRP Substrate Kit; Vector Laboratories; SK-4600) for 20 min, washed with distilled water for 3 min and thereafter counterstained with hematoxylin. The coronal section underwent qualitative analysis by an independent observer blinded to treatment of the animal, utilizing a light microscope (Olympus, x40/x100/x200

**Supplementary Results**

In both groups of animals, a distinct delineation of traumatized area characterized by depletion of cortical neuronal density could be demonstrated. In animals subjected to craniectomy, also some neuronal loss in hippocampal areas (CA1) was seen; In addition, trauma epicenter in animals treated with decompressive craniectomy (CHI + DC + ACZ) demonstrated marked pseudovacuolisation. Contusional changes could be noted in both experimental groups, however CHI+ACZ animals were characterized by some deeper lying hemorrhagic changes involving large areas of the corpus callosum, while in CHI + DC +ACZ animals the contusions were rather circumscribed and located in cortical layers. AQP4 immunostaining demonstrated clear depletion of aquaporin-expressing cells in the lesioned cortex (albeit AQP4 immunoreactivity was reduced more in CHI + ACZ animals). GFAP staining yielded evidence of astroglia loss in trauma epicenter with clear activation of glia cells both in neighboring cortical areas and in deeper lying hippocampal zones, the latter phenomenon was more prominent in CHI + DC + ACZ animals, probably related to the CA1 cell loss.


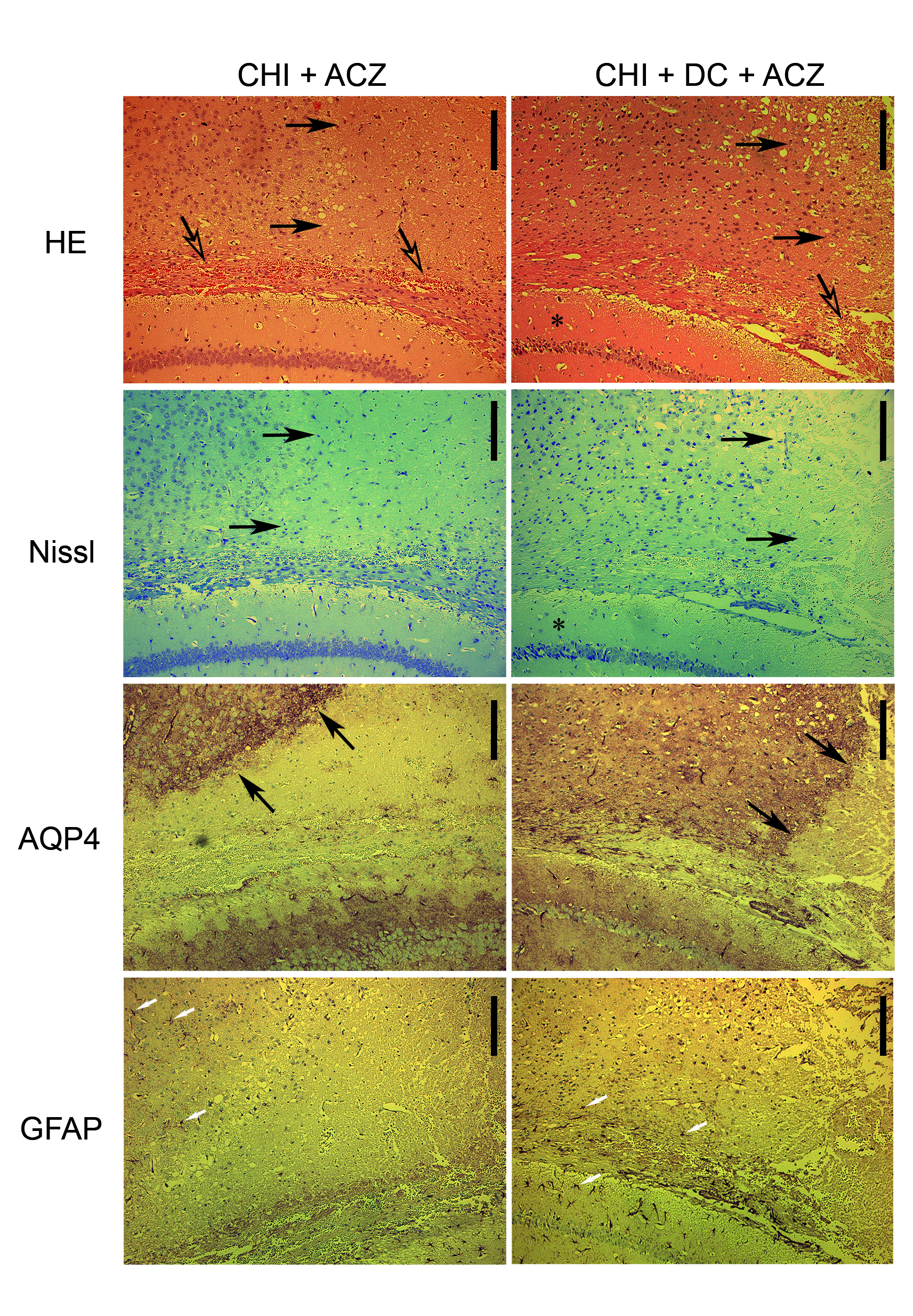


Fig. S1: Demonstrating structural changes in subset of animals subjected to acetazolamide treatment according

to histopathological analysis *(continued on page S3)*

*(continuation)* The upper panel displays photomicrographs of coronal paraffin sections stained with HE and second upper panel demonstrates Nissl-stained sections. Immunohistochemical analysis is represented by AQP-4- (second lower row) and GFAP-immunostaining (lower row).

Arrows demonstrate the border between the trauma epicenter with significant cell loss (both neurons and glia) and between the vital neighboring tissue. Empty arrows are pointing at hemorrhagic changes (diffuse callosal hemorrhage or focal contusion). Asterix marks the area of hippocampal cell loss. White arrows (lower row) demonstrate activated astroglia with strong GFAP immunoreactivity.

Magnification 40x, scale bar: 200 µm.

CHI = closed head injury, DC = decompressive craniectomy, ACZ = acetazolamide.

S3
